# The Current Status of The Demographic and Health Surveys Program

**DOI:** 10.12688/gatesopenres.16400.1

**Published:** 2026-10-14

**Authors:** Madeleine Short Fabic

**Affiliations:** 1 ICF, Rockville, Maryland, 20850, USA

**Keywords:** demographic and health, survey, data access

## Abstract

-->This open letter offers insight into the status of The Demographic and Health Surveys Program and invites readers and other members of the global health community to continue joining in collaborative, future-oriented design and creation for the program’s longevity and evolution.

Since early 2025, individuals and groups—independently and collectively—have been working to ensure that The Demographic and Health Surveys (DHS) Program has both a present and a future. Calls to action have shared the sentiment, “Reinstating the DHS program, or building something better in its place, is both possible and critical” ^1^ with authors of peer-reviewed publications pressing for preservation of long-term pathways to DHS data; incorporation of diverse funding and leadership structures; increased country ownership and oversight; and continuation of international collaboration for accessibility and standardization. ^2^^–^^4^ Similarly, the United Nations has convened a working group, hosted meetings, and written several related reports. ^5^

Building from these efforts, The Demographic and Health Surveys Program continues. Furthermore, the program is actively designing and creating “something better.”

This letter offers insight into the status of The Demographic and Health Surveys Program and invites readers and other members of the global health community to continue joining in collaborative, future-oriented design and creation for the program’s longevity and evolution.

## Stabilization

Demographic and Health Surveys Program surveys are led by the countries in which data are collected, and those data are owned by the countries; this has been the case since the program began in 1984 and is a cornerstone of what makes the program a global good.

As of this writing, with leadership of partner countries, and support from the Gates Foundation, other donors, and the program team at ICF, data from six surveys have been released in the last six months and another 15 surveys are underway. Additional surveys are in planning, as partner countries mobilize funds and determine survey scope.

The DHS Archive, which includes more than 15,000 datasets—alongside related reports, methodological documentation, and more—continues to be publicly available and accessible, as are the Program’s many data user support tools, like STATcompiler, the DHS API, and the *Guide to Statistics.* In the first quarter of 2026, 5,677 users from 124 countries downloaded 452,228 datasets for further analysis. In that same period, the Prime Minister of Ethiopia cited DHS data in his 3 February 2026 remarks to Parliament ^6^; the Government of Kenya highlighted DHS data in its Population Situation Analysis Report ^7^ and related Child Marriage and Other Harmful Practices Policy Brief ^8^; and the United Nations issued a joint press release citing female genital mutilation prevalence estimates largely derived from DHS survey data. ^9^

DHS data continue to be widely collected, analyzed, and used.

## Evolution

Through a three-year grant from the Gates Foundation, The Demographic and Health Surveys Program is in a period of intentional evolution. With an eye to mid-2028 (the end of the grant period) the Program is expeditiously working to enact calls to action and ensure long-term success.

First, the Program is taking action to preserve access to DHS data in perpetuity. When the US Government abruptly shifted its foreign assistance priorities, many feared that the DHS Archive would “go dark.” ^10^ With swift efforts of ICF, followed by the Gates Foundation, that outcome never came to pass. Nonetheless, the vulnerability of the single-source funded, and single-source operated archive must be addressed. In response, the Program is taking a two-pronged approach: a) reaching out to partner countries to ensure they have their DHS data and can access it independent of the archive; and b) building partnerships for archive mirror or back-up site(s) hosting, partnerships designed to be unaffected by swings in external funding.

Second, recognizing the exponential growth in artificial intelligence (AI) capabilities, the Program is harnessing the latest AI advances to streamline processes and reduce survey costs and time. The intention is to develop tools partner countries can use directly; tools that diminish the need for intensive technical assistance. Proof-of-concept testing for three AI tools—to streamline data processing, simplify final report writing, and facilitate data use—has shown promise. The Program is now working to scale these tools and explore additional AI innovations; for example, to streamline and modularize survey questionnaires, and automate the resulting adaptation cascade. All innovations are focused on reducing survey costs, time, and technical assistance; maintaining quality; and reinforcing country ownership.

Third, the Program is actively engaging with partner countries, funders, and other interested parties to identify and forge governance, implementation, and financing structures for the long term.

At the survey level, this includes efforts to make the surveys more modular to better reflect country priorities (governance); to use technological innovations to shrink the need for intensive technical assistance (implementation); and to actively support partner countries’ efforts to fill financing gaps by connecting them with funders, elevating their needs in global fora, and promoting the use of complementary data and small area estimation to minimize demands of the survey that can exacerbate funding gaps (financing).

At the program level, to provide a foundation for governance, implementation, and financing discussions and actions, the Demographic and Health Surveys Program has recently articulated the five key features that collectively make the program a global good: country ownership, accessibility, quality and ethics, comprehensive scope, and standardization ( Table 1). Meanwhile, the program is examining how other “global goods” have been collectively maintained; is participating in the United Nations Task force on sustainable demographic and health statistics through surveys; and is discussing with interested parties far and wide how the global good should be governed (governance), what must be maintained for the global good to continue (implementation), and what financial resources are necessary to ensure the program remains a program rather than a series of one-off surveys (financing).

**Table 1.** Five key features collectively make the DHS program a global good.

Key Features	Components	This feature supports
**Country Ownership**	• Led and implemented by partner country • Incorporated in national systems • Aligned with national priorities • Aligned with or informs international standards • Capacity strengthened for the survey to data-use cycle	• Integrated data systems • Sustainability • Legitimacy • Data use
**Accessibility**	• Data Archive, including related reports • Guide to DHS Statistics • Tools (e.g., STATcompiler, GitHub, Spatial Data Repository), guidance, documentation, and training to support data use	• Public access to microdata + metadata • Discoverability and usability of DHS data and tools • Harmonized integration with other data • Translation of data availability into widespread data use for evidence-informed decision-making
**Comprehensive Scope**	• Comprehensive demographic + health primary data collection • Continuous maintenance of survey tools • Periodic updates to tools + content (e.g., standard questionnaires) • Deliberate, coordinated innovation (e.g., biomarker measurement, GPS masking/displacement, wealth index calculation)	• Comprehensive data support complex analyses to answer pressing population, health, and nutrition questions • Adaptation and consistency meet emerging + existing data needs • Inclusion of new features and technologies expands data access and use, improves quality, and reduces time and cost • Norm-setting + coordinated governance of methodological change
**Quality & Ethics**	• Quality assurance processes + documentation • Independent quality control + technical stewardship that is globally representative • Neutral technical arbiter • IRB approval • Respondent protections, including anonymization	• Reliability • Trust • Replication • Confidentiality • Data integrity • Ethical standards upheld as technologies evolve
**Standardization**	• Standard indicators • Survey methodology • Adaptable standard questionnaires • Standard data editing/weighting • Standard recode files	• Comparability over time and place • Nationally representative data collection • Ease of analysis and aggregation for widespread use • Comprehensive understanding of population, health, nutrition

## Engagement

The Demographic and Health Surveys Program is optimistic about the future. Collective action that builds from existing strengths will create “something better.” With that optimism, the Program shares two invitations:

1) ICF/The DHS Program has issued a Request for Information (RFI): “Establishment of Mirror or Backup Sites for The Demographic and Health Surveys Program Archive.” The RFI is available at dhsprogram.com with responses due by 19 June 2026. Please read, share, and respond. Purposeful partnership will safeguard the DHS Archive for generations to come.
2) ICF, where the DHS Program continues to be housed, recognizes country ownership of surveys and collective ownership of this global good. As a member of the global health community, and active contributor to the creation and maintenance of this global good, ICF deeply cares about ensuring the DHS Program’s continuation, regardless of implementation model or home. In the coming months, the Program will host a series of in-person and virtual events to discuss future directions and opportunities. Please sign up for the DHS Program newsletter to receive related invitations ( https://dhsprogram.com/email-subscribe.cfm). Similarly, as you have ideas and suggestions or host related convenings, please reach out, including by directly emailing the author of this open letter.

In conclusion, the current period of stability and evolution offers the opportunity for collective agency towards creating the next generation program. Calls to action are being heeded. Exciting, positive change is afoot.

## Disclaimer

The views expressed in this article are those of the author. Publication in Gates Open Research or elsewhere does not imply endorsement by the Gates Foundation.

## Data Availability

No data are associated with this article. All DHS Program survey data are available at: https://dhsprogram.com/data/available-datasets.cfm.
